# Tribal warfare: Commensal *Neisseria* kill pathogen *Neisseria gonorrhoeae* using its DNA

**DOI:** 10.15698/mic2019.12.701

**Published:** 2019-10-19

**Authors:** Magdalene So, Maria A. Rendón

**Affiliations:** 1 Department of Immunobiology and the BIO5 Institute, University of Arizona, Tucson, AZ 85721, USA; E-mail: somaggie@email.arizona.edu

**Keywords:** commensal Neisseria, Neisseria gonorrhoeae, DNA killing

## Abstract

It is now abundantly clear that our microbiota (commensals) are critical for many physiological and developmental processes. They have also been shown to inhibit pathogen colonization, through a variety of means including nutrient competition and secretion of microbicidal or biofilm-inhibiting proteins/peptides. Our recent study, Kim *et al.,* (2019), adds a new dimension to the concept of commensal protection. It shows that commensal *Neisseria* kill the closely related pathogen *N. gonorrhoeae* through an unexpected mechanism, one that involves genetic competence, DNA methylation state and recombination. This microreview summarizes the report and discusses questions and lines of research arising from the study. Further investigation into this DNA-based killing mechanism will provide a better understanding of *Neisseria* biology and commensal-pathogen interactions on the mucosa, and identify strategies for preventing pathogenic *Neisseria* transmission.

## GENESIS OF THE STUDY

The Gram negative *Neisseria* genus contains many species that naturally colonize a wide range of animals, including man. Human-dwelling *Neisseria* are genetically related and their chromosomes have large regions of sequence homology and synteny. Only two species in this genus are pathogenic. *Neisseria gonorrhoeae* (Ngo) causes a similarly named sexually transmitted infection and *Neisseria meningitidis* (Nme) causes septicemia and meningitis, both in man. Not surprisingly, they have been the focus of attention of researchers and funding agencies, and the commensal species have largely been ignored. A microbiome study of 242 healthy human subjects revealed that *Neisseria* are abundant on all 18 body sites sampled. As Ngo infects some of these niches, we asked a simple question: What happens when this pathogen enters an environment containing a community of commensal *Neisseria*?

## COMMENSAL *NEISSERIA* KILL PATHOGENIC *NEISSERIA* VIA ITS DNA

We found that Ngo is killed in the presence of the human commensal *Neisseria elongata* (Nel), and the toxic compound is Nel DNA that accumulated in the medium. Ngo competence mutants resist killing by Nel DNA. *In vivo* results are consistent with these findings. In a mouse model of lower genital tract infection, Nel accelerates Ngo clearance from the vagina and a Ngo DNA uptake mutant resists this clearance. Any DNA will kill, provided that it enters Ngo, its methylation pattern is foreign to the pathogen, and it has homology to the pathogen chromosome.

**Figure 1:**
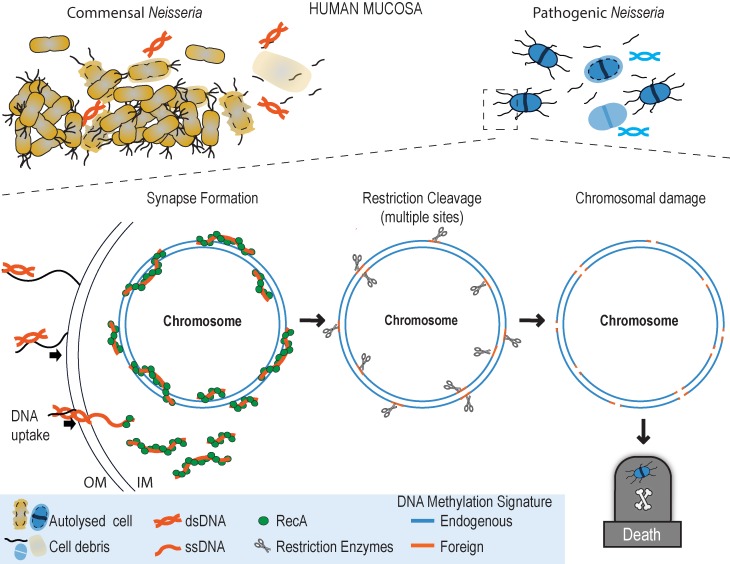
FIGURE 1: Working model for how commensal *Neisseria* DNA kills *Neisseria gonorrhoeae* on the human mucosa (see text for details). Top left: commensal *Neisseria* cells on a mucosal passage. Top right: *Neisseria gonorrhoeae* arriving in the vicinity of the commensal biofilm. Middle: cutaway showing commensal DNA entering a *Neisseria gonorrhoeae* cell, RecA-mediated recombination of this DNA with the pathogen chromosome, restriction enzyme cleavage of the synaptic joint, and loss of chromosome integrity and cell death. Keys to the figures are in the bottom panel.

How does DNA kill Ngo? Ngo takes up DNA released from autolysed *Neisseria* cells, using the Type IV pilus (Tfp)-based system that specifically binds *Neisseria* DNA. DNA uptake is highly efficient: over 10% of cells are competent for transformation. Evidence from *Neisseria* and other bacteria indicate that DNA entering the cell becomes single stranded as it crosses the cell wall. Other studies show RecA quickly binds single stranded DNA in the cytoplasm and facilitates its recombination with a homologous sequence in the bacterial genome. Our study shows that Ngo with a silenced *recA* is resistant to DNA killing. These and other findings led us to propose the following model for the DNA killing mechanism **(Figure 1)**. RecA binds single stranded commensal DNA as it enters the Ngo cytoplasm and initiates the formation of a synaptic joint in the chromosome. In this structure, a strand of commensal DNA is paired with its complementary sequence in the Ngo genome. As the DNA methylases of Nel and Ngo have different specificities, the two strands in this heteroduplex would have different methylation signatures.

As commensal DNA altered to mimic the Ngo methylation pattern is less toxic to Ngo, we propose that Ngo restriction enzyme(s) cleave at cognate sequences in these heteroduplexes. One or a few double-strand breaks in the chromosome will likely be repaired quickly by resident repair enzymes. However, Ngo produces numerous Tfp fibers, many of which are likely to be actively taking up DNA. Internalized segments from different regions of the commensal chromosome would, through RecA, form synaptic joints with multiple chromosomal sites. We propose that restriction cleavage of large numbers of synapses overwhelms repair enzymes, compromising chromosome integrity and, by implication, cell viability.

## IMPLICATIONS OF OUR STUDY

As with unexpected findings, ours raises many questions and suggests lines of investigation. At the molecular level, we need to assess the extent of synapse formation following DNA uptake. Knowing the amount of commensal DNA that Ngo takes up concurrently, the sequence complexity of this DNA, and restriction enzyme activity on heteroduplexes with different methylation signatures will allow us to do this.

At the organismal level, we need to better understand the processes of autolysis, DNA extrusion and DNA uptake. Commensal and pathogenic *Neisseria* undergo autolysis, releasing DNA that is transformable. Approximately 4% of Ngo cells in a culture undergo autolysis; the frequency of autolysis in commensal species needs to be evaluated. For reasons unknown, Ngo also actively extrudes its DNA. DNA uptake likely plays a primary role in horizontal gene transfer among *Neisseria*, including the acquisition of antibiotic resistance alleles by Ngo (see below), because this genus is not known to acquire DNA by conjugation (except for conjugative plasmids described for Ngo) and there are few reports of phages in *Neisseria*. Are autolysis, DNA extrusion and DNA uptake regulated in *Neisseria*? If so, what physiological conditions trigger their activation? Delving into these questions will provide clarity on commensal/pathogen encounters at the mucosal surfaces and clues to *Neisseria* microevolution.

A few Ngo cells survive DNA killing. A host of literature predicts that some of these survivors do not express Tfp. When Ngo is not piliated, it takes up DNA at a markedly lower frequency. As predicted, our preliminary results suggest that some survivors do not produce Tfp. However, some do. How do these cells escape DNA killing? Are survivors proficient in colonization (*in vitro* and *in vivo*)?

Why, one might ask, would Ngo express such a highly active DNA uptake system if this increases its chances of being killed by commensal DNA? We speculate that the efficiency of the system likely reflects the need of the pathogen to balance the pressures imposed by antibiotic and commensal DNA killing, perhaps also by other elements that challenge its survival.

Another question arising in conversation is whether Ngo returns the favor, and kills commensal *Neisseria* with its DNA. Our study examined only Nel; in this case, neither Ngo cells nor Ngo DNA kills the commensal at a detectable frequency. We did not pursue this line of investigation, but can offer several possible explanations for this observation. Nel produces few Tfp and transforms at a much lower frequency compared to Ngo; moreover, Nel has fewer restriction/modification (R/M) systems. Our *in silico* analysis showed that other commensal *Neisseria* species also have fewer R/M loci, but we did not validate these findings. So whether Ngo kills other commensal *Neisseria* species remains to be tested. An interesting corollary to this line of thought is whether commensal *Neisseria* use the DNA killing mechanism to jostle for dominance on the mucosal surfaces.

## CONNECTING OUR FINDINGS TO CLINICAL OBSERVATIONS

Ngo is considered a relatively weak pathogen as only 20-70% of infected individuals develop signs of disease. Pathogenicity is likely multifactorial, but the DNA killing mechanism and the presence/abundance of commensal *Neisseria* at the infection site (we know nothing about the biogeography of *Neisseria* in the body) are likely to be important determinants. It has been known for many years that the presence of commensal *Neisseria lactamica* (Nla) in the upper respiratory tract reduces the risk of meningococcal disease. How Nla protects against Nme infection is unknown, but our study provides a mechanistic link: we found that Nla DNA kills Nme.

## HOW WIDESPREAD IS THE DNA KILLING MECHANISM?

Several mucosal pathogens, including *Streptococci* and *Haemophilus influenzae*, are genetically competent and the latter has its own dedicated DNA uptake system. Do these bacteria use a similar DNA-based mechanism to establish and/or maintain their niche?

## TRANSLATIONAL APPLICATION

Although our study began with a basic science question, it may nevertheless have translational potential. The global incidence of gonorrhea has risen steadily and the World Health Organization estimates that in 2018 alone there were nearly 100 million new cases of gonorrhea. Since the 1930s, a succession of antibiotics has been used to treat gonorrhea, but they were discontinued one by one as Ngo developed resistance to them. Multidrug resistant and extremely drug resistant Ngo have been appearing since the 1990s. With this pathogen becoming resistant to the last line of antibiotics and with no new compounds in development, experts predict gonorrhea will soon be untreatable. Efforts to develop a gonococcal vaccine are ongoing. At this juncture, public health organizations have begun to consider nontraditional modalities for countering Ngo infection.

Our observation that any suitably modified DNA will kill Ngo suggests one straightforward strategy for blocking its transmission: using DNA to kill Ngo acquired during sexual contact, for instance by incorporating it into vaginal gels/lubricants. Experiments are under way to test this idea and to engineer DNA to kill Ngo specifically while leaving commensal *Neisseria* unharmed.

